# The physiological impact of high‐intensity interval training in octogenarians with comorbidities

**DOI:** 10.1002/jcsm.12724

**Published:** 2021-05-31

**Authors:** James E.M. Blackwell, Nima Gharahdaghi, Matthew S. Brook, Shinya Watanabe, Catherine L. Boereboom, Brett Doleman, Jonathan N. Lund, Daniel J. Wilkinson, Kenneth Smith, Philip J. Atherton, John P. Williams, Bethan E. Phillips

**Affiliations:** ^1^ MRC‐Versus Arthritis Centre for Musculoskeletal Ageing Research, Royal Derby Hospital Centre University of Nottingham Derby UK; ^2^ National Institute of Health Research (NIHR) Nottingham Biomedical Research Centre (BRC) Nottingham UK; ^3^ Department of Surgery & Anaesthetics Royal Derby Hospital Derby UK

**Keywords:** HIIT, Exercise, Disease, Ageing, Muscle, Protein synthesis

## Abstract

**Background:**

Declines in cardiorespiratory fitness (CRF) and fat‐free mass (FFM) with age are linked to mortality, morbidity and poor quality of life. High‐intensity interval training (HIIT) has been shown to improve CRF and FFM in many groups, but its efficacy in the very old, in whom comorbidities are present is undefined. We aimed to assess the efficacy of and physiological/metabolic responses to HIIT, in a cohort of octogenarians with comorbidities (e.g. hypertension and osteoarthritis).

**Methods:**

Twenty‐eight volunteers (18 men, 10 women, 81.2 ± 0.6 years, 27.1 ± 0.6 kg·m^−2^) with American Society of Anaesthesiology (ASA) Grade 2–3 status each completed 4 weeks (12 sessions) HIIT after a control period of equal duration. Before and after each 4 week period, subjects underwent body composition assessments and cardiopulmonary exercise testing. Quadriceps muscle biopsies (*m. vastus lateralis*) were taken to quantify anabolic signalling, mitochondrial oxidative phosphorylation, and cumulative muscle protein synthesis (MPS) over 4‐weeks.

**Results:**

In comorbid octogenarians, HIIT elicited improvements in CRF (anaerobic threshold: +1.2 ± 0.4 ml·kg^−1^·min^−1^, *P* = 0.001). HIIT also augmented total FFM (47.2 ± 1.4 to 47.6 ± 1.3 kg, *P* = 0.04), while decreasing total fat mass (24.8 ± 1.3 to 24 ± 1.2 kg, *P* = 0.0002) and body fat percentage (33.1 ± 1.5 to 32.1 ± 1.4%, *P* = 0.0008). Mechanistically, mitochondrial oxidative phosphorylation capacity increased after HIIT (i.e. citrate synthase activity: 52.4 ± 4 to 67.9 ± 5.1 nmol·min^−1^·mg^−1^, *P* = 0.005; membrane protein complexes (C): C‐II, 1.4‐fold increase, *P* = 0.002; C‐III, 1.2‐fold increase, *P* = 0.03), as did rates of MPS (1.3 ± 0.1 to 1.5 ± 0.1%·day^−1^, *P* = 0.03). The increase in MPS was supported by up‐regulated phosphorylation of anabolic signalling proteins (e.g. AKT, p70S6K, and 4E‐BP1; all *P* < 0.05). There were no changes in any of these parameters during the control period. No adverse events were reported throughout the study.

**Conclusions:**

The HIIT enhances skeletal muscle mass and CRF in octogenarians with disease, with up‐regulation of MPS and mitochondrial capacity likely underlying these improvements. HIIT can be safely delivered to octogenarians with disease and is an effective, time‐efficient intervention to improve muscle mass and physical function in a short time frame.

## Introduction

Sarcopenia is characterized by a decline in skeletal muscle mass and function and is common to ageing.^1^ Reflecting this, the impact of sarcopenia on older people is far reaching, leading to frailty, loss of mobility, increased risk of chronic diseases, and poorer clinical outcomes if hospitalization is required.^2^, ^3^ In addition, cardiorespiratory fitness (CRF) also declines with ageing,^4^ and lower anaerobic threshold (AT), an indicator of CRF, is associated with worse clinical outcomes in individuals with comorbidities.^5^ Together, these maladaptations are inextricably linked to numerous detrimental health outcomes in the older adult.^6^ Further, by 2050, the number of adults over the age of 85 years will triple,^1^ as will the prevalence of age‐associated comorbidities (e.g. cardiovascular disease, hypertension, cancer, osteoarthritis, diabetes mellitus, osteoporosis and mobility disability^7^) and mortality risk.^4^ On this basis, there remains a critical need to identify therapeutic interventions to counteract age‐associated changes on the background of multi‐comorbidities.

Exercise is an optimal strategy to enhance both muscle mass and function^1^ and CRF^4^ in older adults. However, these two physiological adaptations are best achieved through different modes of exercise (i.e. resistance exercise training and endurance exercise training, respectively), each of which requires a significant time commitment and training duration to achieve meaningful benefit.^8^ As a time‐efficient alternative, high‐intensity interval training (HIIT; encompassing brief periods of high‐intensity exertions interspersed with periods of rest or active recovery^8^, ^9^) has emerged as a training strategy with potential to improve both muscle mass and CRF in both younger healthy individuals^9^, ^10^, ^11^ and individuals with specific diseases (e.g. cardiovascular disease,^2^ cancer,^3^ and obesity^9^). In addition, in recent years, HIIT has also been shown to elicit benefits in healthy older adults.^12^ However, these studies have mainly focused on ‘younger’ older adults (∼70 years) and not the oldest old individuals with diseases, potentially lacking relevance to large swathes of the population with severe functional performance decrements.^1^ In addition, disuse periods, which are more frequent in octogenarians (due to hospitalization/institutionalization), result in further declines in muscle mass and function, placing these oldest old individuals at higher risk of intrinsic capacity declines (e.g. physical and cognitive impairments) with subsequent lower adaptability to exercise.^1^ However, to date, the efficacy of HIIT as an exercise training mode in octogenarians with comorbidities is poorly defined.

The mechanisms by which HIIT enhances muscle mass and improves CRF have been subject to intense study. For instance, HIIT‐induced activation of the phosphoinositol‐3 kinase‐Akt–mTOR signal transduction pathway led to higher ribosomal biogenesis and muscle protein synthesis (MPS) and induced hypertrophy to a greater extent than moderate intensity training.^13^ Further, just a single session of HIIT was shown to increase MPS, which remained elevated for up to 48 h, in healthy older men.^14^ In addition to the detrimental impact of advancing age on cellular processes associated with MPS,^1^ proteins involved in the regulation of mitochondrial biogenesis and the electron transport chain have also been found to be down‐regulated in older adults resulting in reduced capacity for cellular bioenergetics^15^ and associated organ system functions (i.e. CRF^16^). Reflecting this, HIIT for 6 weeks was shown to increase mitochondrial content and respiratory capacity in older adults.^15^ Furthermore, 8 weeks of HIIT, but not work‐matched continuous moderate intensity training, increased mitochondrial respiration in younger adults.^17^ Six weeks HIIT also increased mitochondrial content as assessed by citrate synthase (CS) activity^18^ and mitochondrial oxidative capacity as assessed by the abundance of mitochondrial proteins and their functional capacity to produce ATP,^18^ resulting in increased bioenergetic efficiency of muscle.^19^

In summary, neither the efficacy nor mechanisms of HIIT in respect to inducing favourable physiological adaptations in ‘very’ old adults with disease (i.e. comorbid octogenarians) are well‐established, despite this group representing a large proportion of our aged societies. As such, this study aimed to evaluate the impact of 4 weeks HIIT on CRF and body composition changes in octogenarians bearing diseases, while mechanistically reporting endpoints relating to mitochondrial adaptations, anabolic signalling, and MPS.

## Methods

### Ethics and recruitment

This study was approved by the University of Nottingham Faculty of Medicine and Health Sciences Research Ethics Committee (A14072015‐SoM‐MSGEM), was conducted according to the Declaration of Helsinki, and was pre‐registered online (clinicaltrials.gov; NCT03138265). Participants were recruited from community groups in close geographical proximity to the study site. Before entry into the study, participants provided written informed consent to participate after all procedures, and risks were explained to them. A medical practitioner screened all participants by medical questionnaire, physical examination, routine blood haematology/biochemistry, resting blood pressure, cardiorespiratory examination, and a resting electrocardiogram. Inclusion criteria were independent community‐dwelling individuals aged over 75 years and American Society of Anaesthetists (ASA) physical status classification system Grade 2 or 3 (Grade 2 = a patient with mild systemic disease; Grade 3 = a patient with severe systemic disease).^20^ ASA grading is used clinically to assess the preoperative health of surgical patients based on five classes from 1 (*Patient is a completely healthy fit patient*) to 5 (*A moribund patient who is not expected to live 24 h with or without surgery*). In this study, ASA grading was assigned by a blinded consultant anaesthetist. Volunteers were required to have had no change to regular medications in the month prior to or during the study period; be available for the entire study period; and be physically able to cycle on a static cycle ergometer. Participants were excluded based on the American Thoracic Society/American College of Chest Physicians (ATS/ACCP) cardiopulmonary exercise test (CPET) guidelines^21^; or if they were already undertaking >2 exercise sessions per week.

### Study conduct

**Figure 1:**
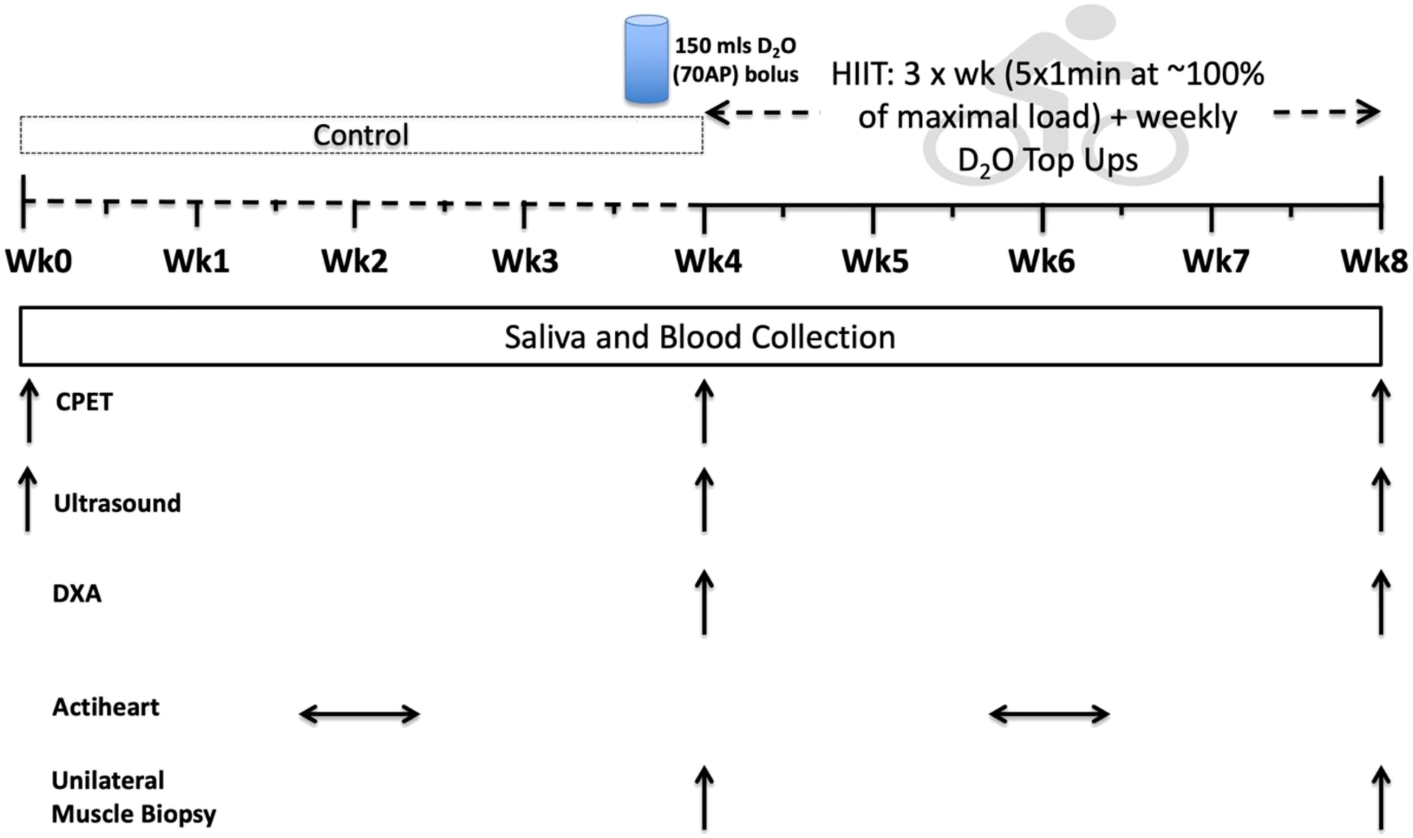
Schematic representation of the study protocol. Abbreviations: CPET, cardiopulmonary exercise test; DXA, dual‐energy x‐ray absorptiometry; HIIT, high‐intensity interval training. HIIT, 3× each week at ∼100% of maximal load from the second CPET.

Following baseline measurements, a no‐intervention control period of 4 weeks (CON; 28.8 ± 5.5 days) was followed by second assessment session. Participants then completed 4 weeks of HIIT (29.3 ± 3.8 days) before a final (i.e. third) assessment session (48–72 h after last session of HIIT session) (*Figure* 1). This single‐group study design with a control lead‐in period was used to ensure that no data sets were influenced by changes in habitual physical activity [or other behavioural influences (e.g., diet)] occurring secondary to study participation alone. Participants were asked to maintain their habitual dietary intake for the duration of the study.

Each assessment session involved collection of a blood sample, muscle ultrasound of the *m. vastus lateralis* (VL), a CPET, and assessment of resting heart rate and blood pressure.^22^ A dual‐energy X‐ray absorptiometry (DXA; Lunar Prodigy II, GE Medical Systems, Little Chalfont, UK) scan was performed after the CON and HIIT periods. Participants wore a tri‐axial physical activity monitor (Actiheart, CamMtech Ltd, Cambridge, UK) for a 5 day period around the mid‐point of both CON and HIIT periods. In order to assess rates of MPS throughout each period (*n* = 20), a basal (rested/fasted) saliva sample was collected at each assessment session, with a VL muscle biopsy collected at assessment Sessions 2 and 3 from a subset of participants who volunteered to have biopsies. At assessment Session 2, a single dose (150 mL) of D_2_O was consumed after saliva collection and a muscle biopsy. The initial priming dose of D_2_O was followed by weekly small‐volume ‘top‐ups’ of ∼50 mL (calculated from measures of each individual's body water pool turnover). Daily saliva samples were also collected for 3 days after each D_2_O dose. All muscle samples were collected under sterile conditions using the conchotome biopsy technique,^23^ with 1% w·v^−1^ lidocaine as local anaesthetic. Any fat and connective tissue was rapidly dissected out and muscle was washed in ice‐cold phosphate‐buffered saline, blotted dry and frozen in liquid nitrogen, before storage at −80°C.

#### High‐intensity interval training sessions

Each HIIT session lasted 16.5 min (*Figure* 1) with participants completing a total of 12 sessions (three sessions/week for 4 weeks). The HIIT protocol was performed on a cycle ergometer (Lode Corival, Lode, Groningen) and comprised a 2 min warm‐up period of unloaded cycling, followed by five, 1 min exertions at 100–115% of the maximal load (watts, W) reached during their initial CPET, separated by 90 s unloaded cycling, and ending with a 2 min recovery period of unloaded cycling.^24^ Intensity during the exertions ranged from 46 to 226 W, with men exercising at a significantly higher intensity than women (131 ± 37 vs. 82 ± 25 W, *P* = 0.003). For participants reporting a rating of perceived exertion of eight or less (on a 0–10 scale) at the end of the last interval at the mid‐way point of training (71% of participants), an (10%) increase in wattage was implemented to maintain exercise intensity with progression.^24^

### Assessment measures

#### Cardiopulmonary exercise testing

A CPET was conducted in accordance with the ATS/ACCP joint statement on CPET.^21^ In brief, using a 10–20 W/min ramp protocol (based on gender, height, weight, and self‐reported habitual physical activity) on a Lode Corival cycle ergometer (Lode Corival, Lode, Groningen, The Netherlands) with inline gas analyser (ZAN 680, nSpire Health, Colorado, USA), participants initially rested for 3 min followed by 2 min unloaded cycling as a warm‐up. For the duration of the test, participants were instructed to pedal at 55 revolutions per min (rpm) and to continue until volitional exhaustion. Tests were considered maximal if three or more of the following criteria were met: (i) a plateau in the O_2_ uptake curve despite rising *V*CO_2_; (ii) a respiratory exchange ratio of >1.1; (iii) a heart rate over 85% age‐predicted maximum, and (iv) a rating of perceived exertion ≥9 using a modified (0–10) Borg scale.^25^ Participants were monitored throughout with a 12‐lead electrocardiography, pulse oximetry, and non‐invasive blood pressure monitoring. All CPET sessions were overseen by an advanced life support‐trained clinician with termination criteria taken from the ATS/ACCP statement.^21^ Values obtained from the CPET included: VO_2_ peak (volume of oxygen consumed at maximal exertion); AT: volume of oxygen consumed at AT, determined and analysed using a modified V‐slope and ventilatory equivalents method^24^, and maximal and sub‐maximal heart rate parameters.^24^

#### Muscle mass and architecture

B‐mode ultrasonography (MyLab 70, Esaote Biomedica) with a 100 mm, 10–15 MHz linear array probe was used for quantification of myo‐architecture. Measures of muscle thickness (MT), pennation angle (PA) and fascicle length (FL) were made^26^ at 50% of the VL length (halfway between the greater trochanter and midpoint of the patella), with ImageJ software (ImageJ 1.51 h) used to analyse the images. DXA was used to determine total fat‐free mass (FFM), total fat percentage (TFP), total fat mass (TFM), and FFM index:

$$\mathrm{FFM}\;\left(\mathrm{kg}\right)/\text{height}\;{\left(m\right)}^{2}$$

before and after the HIIT period using our standard DXA protocol.^26^

#### Muscle protein synthesis

The saliva samples collected were processed to determine each participant's body water enrichment over the time between biopsies, to provide a measure of the precursor labelling, as described previously.^27^ In brief, 80–90 μL of saliva was heated for 4 h at 90–100°C and then cooled on ice to produce a condensate of the body water. A high‐temperature conversion elemental analyser (Thermo Finnigan, Thermo Scientific, Hemel Hempstead, UK) connected to an isotope ratio mass spectrometer (Delta V advantage, Thermo Scientific) was employed to measure deuterium labelling in the water (0.1 μL).

To assess protein bound alanine muscle fraction enrichment, ∼40 mg of muscle was homogenized in ice‐cold homogenization buffer to isolate myofibrillar proteins.^28^ Briefly, 10 min rotary mixing was followed by centrifugation at 11 000 *g* for 15 min at 4°C. The supernatant (sarcoplasmic fraction) was then collected for immunoblotting and the pellet was re‐suspended in 500 μL mitochondrial extraction buffer and then homogenized by Dounce, and centrifuged at 1000 *g* for 5 min at 4°C. Insoluble collagen was separated following centrifugation from myofibrillar proteins that were solubilized in 750 μL NaOH and subsequently precipitated using 1 M perchloric acid then pelleted by centrifugation. For the plasma proteins (used to represent baseline deuterium labelling in protein), 100 μL of sample was precipitated using 100 μL ice cold ethanol and then separated from free amino acids through centrifugation. Muscle and plasma protein extracts, were hydrolyzed overnight in 0.1 M HCl and Dowex H^+^ resin at 110°C, before elution of the protein‐bound amino acids, from the resin with 2 M NH4OH then evaporated to dryness.^28^ Dried samples were suspended in 60 μL distilled water, 32 μL methanol, and 10 μL of pyridine, and 8 μL of methyl chloroformate with intermittent vortex. The *n*‐methoxycarbonyl methyl esters of the amino acids were then extracted after adding 100 μL chloroform. A molecular sieve was added for ∼20 s to remove water before being transferred to vials; incorporation of deuterium into the protein bound alanine was determined by gas chromatography‐pyrolysis‐isotope ratio mass spectrometry (Delta V Advantage, Thermo, Hemel Hempstead, UK).^28^

#### Calculation of synthetic fractional rate

Myofibrillar MPS was calculated from the deuterium enrichment (APE) in alanine in myofibrillar proteins, using the body water enrichment, from saliva (APE, corrected for the mean number of deuterium moieties incorporated per alanine, 3.7, and the dilution from the total number of hydrogens in the derivative, i.e. 11) as the average precursor labelling between subsequent muscle biopsies. The fractional synthetic rate was calculated as follows:

$$\mathrm{FSR}\left(\%\cdot {\mathrm{day}}^{-1}\right)=-\text{In}\left(\frac{1-\left[\frac{({\mathrm{APE}}_{\mathrm{Ala}}}{{\mathrm{APE}}_{P}}\right]}{t}\right),$$

where APE_Ala_ is deuterium enrichment of protein‐bound alanine, APE_P_ is mean precursor enrichment of the body water over the period, and *t* is the time between biopsies. Absolute synthetic rate was estimated as

$$\mathrm{ASR}\left(g\cdot {\mathrm{day}}^{-1}\right)=\frac{\mathrm{FSR}}{100}\times \text{Total}.\mathrm{FFM}\times \frac{12.4}{100},$$

where alkali soluble protein of 12.4% total FFM was assumed.^28^

#### Immunoblotting for anabolic signalling and mitochondrial oxidative phosphorylation

To prepare samples for immunoblotting, spectrophotometry was used to determine protein concentrations of sarcoplasmic fractions and samples were diluted with 3× Laemmli loading buffer to 1 mg·mL^−1^, followed by heating at 95°C for 5 min.^29^

To measure mitochondrial oxidative phosphorylation (OxPhos), after homogenization of ∼10 mg muscle in radioimmunoprecipitation assay buffer (50 mM Tris HCl, PH 7.4, 150 mM NaCl, 1% Triton X‐100, 0.5% sodium deoxylcholate, 0.1% sodium dodecyl sulfate (SDS)) followed by quick vortex and 10 min sonication, samples were centrifuged at 6000 *g* for 3 min, and then supernatant was used for protein concentration determination using Pierce™ BCA Protein Assay Kit (23225, Thermo Scientific). Samples were diluted with 3× Laemmli loading buffer to 0.5 mg·mL^−1^, followed by heating at 40°C for 5 min.

For the measurements of anabolic signalling proteins and OxPhos, precisely 10 μg of samples were loaded onto Criterion XT Bis–Tris–12% SDS‐polyacrylamide gel electrophoresis (SDS‐PAGE) gels (Bio‐Rad) for electrophoresis at 185 V for 45 min. After electrophoresis, samples were transferred onto polyvinylidene difluoride membranes for 45 min at 100 V. Subsequently, 2.5% low‐fat milk, which was diluted in Tris‐buffered saline Tween‐20 was used to soak and block polyvinylidene difluoride membranes for 1 h, at ambient temperature and then incubated in the following primary antibodies overnight at 4°C (1:2000 dilution in 2.5% BSA in TBS‐T): rabbit phospho‐protein kinase B (Akt)^Ser473^ (#9271), phospho‐p70 S6 Kinase (p70S6K)^Thr389^ (#9234), phospho‐4E‐BP1^Thr37/46^ (#2855), phospho‐AMP‐activated protein kinase (AMPKα)^Thr172^ (#2531), phospho‐forkhead box O3 (FoxO3a)^Ser253^ (#13129), phospho‐tuberin/TSC2^Thr1462^ (#3617) (all from Cell Signalling Technology, Leiden, The Netherlands), muscle‐specific F‐box protein (MAFbx) (#AP2041), muscle RING‐finger protein‐1 (MURF‐1) (#101AP) (both from ECM Biosciences, Versailles, KY, USA), mouse oxidative phosphorylation (OxPhos) (ab110413, Abcam, Cambridge, MA, USA). After overnight incubation, membranes were washed for 3 × 5 min in TBS‐T, soaked in horseradish peroxidase (HRP)‐conjugated secondary antibody (New England Biolabs; 1:2000 in 2.5% bovine serum albumin (BSA) in Tris‐buffered saline Tween‐20) for 1 h, before 3 × 5 min washes in TBS‐T. In order to quantify band intensity (Chemidoc MP, Bio‐Rad, Hemel Hempstead, UK), membranes were exposed to Chemiluminescent HRP substrate (Millipore Corp., Billerica, MA, USA) for one min. Relative arbitrary units (AU) were normalized to coomassie‐stained membranes and to cross gel loading control.^29^

#### Mitochondrial citrate synthase activity

Citrate synthase (CS) activity was measured as previously described.^30^ Briefly, after homogenization of 3–5 mg muscle in 1% Triton‐X‐100 buffer, samples were centrifuged at 22 000 *g* for 3 min and the supernatant used for further analysis. Thereafter, 300 μL master mix containing 28% 0.05 M TRIS buffer (pH 7.6), 1.3% one mM 5,5′ dithiobis‐2‐nitrobenzoic acid (DTNB), 7% acetyl‐coenzyme A (1.36 mg·mL^−1^), 0.8% oxaloacetate (9.88 mg·mL^−1^), and 63% ddH_2_O was measured at 412 nm as the blank. Finally, 20 μL of separated supernatant was used to measure the maximum rate of reaction (V max), compared with whole protein content.

#### Qualitative data

In addition to our physiological endpoints, at each assessment session participants completed the Dukes Activity Score Index (DASI) questionnaire^31^ to assess subjective functional status and the EuroQoL Group EQ‐5D‐5L questionnaire (EQ‐5D‐5L) to assess health status.^32^ Participants also completed a HIIT acceptability questionnaire as used previously^24^ at assessment Session 3 (Supporting information, *Table* S1).

### Statistical analysis

Assuming an 18% coefficient of variation,^24^ to detect a mean clinically significant increase in AT of 2 mL·kg^−1^·min^−1^ following HIIT,^6^ with 1‐β = 0.8 and α = 0.05 significance, we required 28 complete data sets; this was achieved. Data are expressed as mean ± SEM; and normality of data was tested using D'Agostino and Pearson omnibus test. Repeated measure ANOVA with one factor (time) were used to compare the changes during CON and HIIT periods. Paired Student's *t* tests were used to test pre vs. post HIIT changes. Correlations were assessed using Pearson's product moment correlation coefficient. The significance level was defined as *P* ≤ 0.05, and all statistical analyses were performed using GraphPad Prism 8.4.3 (La Jolla, CA, USA).

## Results

### Participants and HIIT

Twenty‐eight participants completed the study (physiological characteristics and comorbidities outlined in *Table* 1). Mean heart rate as a percentage of age‐predicted maximum^33^ was 96.6%, 95.7%, and 94.8% during HIIT Sessions 2, 6, and 10, respectively, confirming high‐intensity exercise was being performed.^33^ Participants completed an average of 11.5 ± 0.9 sessions (range 11–12) out of the 12 available.

**Table 1:** Participant characteristics and comorbidities

Age (years)		81.2 (0.6)
Gender (men: women)		18: 10
BMI (kg·m^−2^)		27.1 (0.6)
ALMI (kg·m^−2^)		7.4 (0.1)
ASA Grade (2:3)		21:7
SBP		150 (3.2)
DBP		79 (1.7)
Resting HR		67 (2)
VO_2_ peak (mL/kg/min)		22.1 (1.2)
VO_2_ AT (mL/kg/min)		13.4 (0.5)
Dominant handgrip strength (kg)		27.4 (8.7)
SPPBT score		9.8 (2)
DASI		41.2 (11)
Comorbidities and medications		
System	Pathology	Number
Cardiovascular	Hypertension	20
	Ischaemic heart disease	6
	Heart failure	2
	Valvular disease	2
	Pacemaker	1
	Abdominal aortic aneurysm	1
Respiratory	COPD	1
	Asthma	2
	Previous lung TB	2
Endocrine	Thyroid disorders	4
	Diabetes mellitus	5
	Dyslipidaemia	5
	Gout	2
Musculoskeletal	Osteoarthritis	8
	Large joint replacement	6
Obesity	BMI > 25 (overweight)	14
	BMI > 30 (obese Class 1)	6
Regular medications	Inhaled bronchodilators	3
	Statins	13
	Calcium channel blockers	11
	Beta‐blockers	3
	Amiodarone/digoxin	2
	ACE‐inhibitors/AT1/2 antagonists	7
	Diuretics	7
	Metformin	2
	Thyroxine	4

Abbreviations: ALMI, appendicular lean mass index [sum of the lean tissue in arms and legs (kg) divided by height (m)^2^]; ASA, American Society and Anaesthetists; AT, anaerobic threshold; BMI, body mass index; COPD, chronic obstructive pulmonary disease; DASI, Dukes Activity Status Index; DBP, diastolic blood pressure; HR, heart rate; SBP, systolic blood pressure; SPPBT, short physical performance battery tests; TB, tuberculosis.

Values are mean (SEM).

### Cardiorespiratory fitness

**Figure 2:**
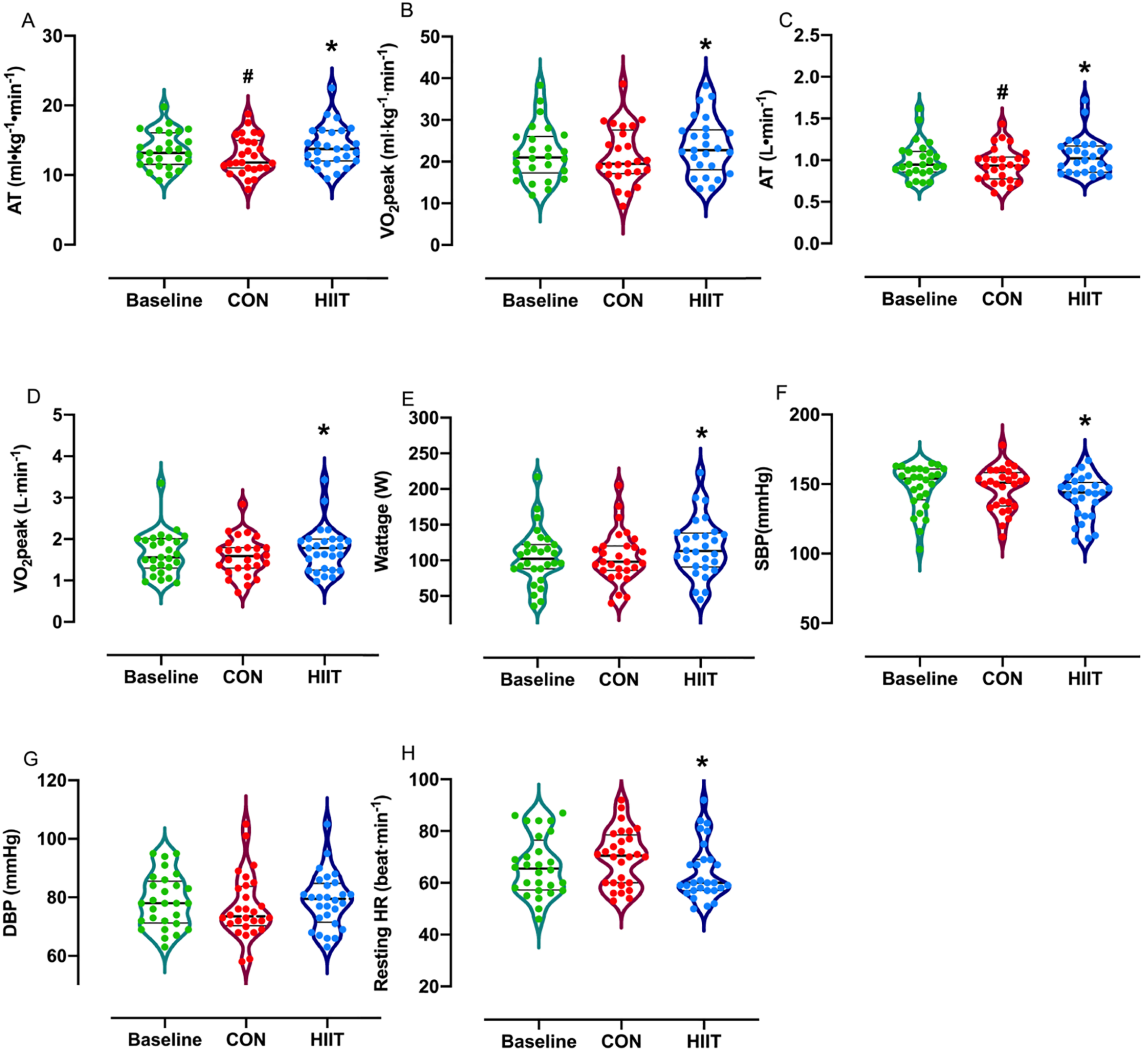
Violin plots represent a full distribution of raw data (dots), median (central line), and interquartile range (lower and upper lines) of cardiorespiratory parameters at baseline, after a control period (CON), and after high‐intensity interval training (HIIT). Abbreviations: AT, anaerobic threshold; DBP, diastolic blood pressure; HR, heart rate; SBP, systolic blood pressure. Analysis via repeated measure ANOVA. * *P* < 0.01 vs. CON, #*P* < 0.01 vs. baseline.

AT (0.94 ± 0.03 to 1.1 ± 0.04 L·min^−1^, *P* = 0.001; *Figure* 2C) and VO_2_ peak (1.6 ± 0.1 to 1.7 ± 0.1 L·min^−1^, *P* = 0.008; *Figure* 2D), both absolute and relative to bodyweight (AT: 12.8 ± 0.5 to 14.1 ± 0.5 mL·kg^−1^·min^−1^, *P* = 0.008; *Figure* 2A, VO_2_ peak: 21.3 ± 1.3 to 23.7 ± 1.3 mL·kg^−1^·min^−1^, *P* = 0.009; *Figure* 2B) increased significantly with HIIT; with a decrease in both absolute and relative measures of AT over CON (vs. the first assessment, absolute: 1.0 to 0.94 L·min^−1^, *P* = 0.005; *Figure* 2C, relative: 13.5 ± 0.5 to 12.8 ± 0.5 mL·kg^−1^·min^−1^, *P* = 0.01; *Figure* 2A). HIIT also elicited significant increases in maximum wattage achieved at CPET (105.1 ± 7 to 117.7 ± 8 W, *P* < 0.0001; *Figure* 2E) and reductions in systolic blood pressure (147 ± 3 to 139 ± 3 mmHg, *P* = 0.001; *Figure* 2F) and resting heart rate (69.8 ± 2 to 64.4 ± 2 beat·min^−1^, *P* = 0.001; *Figure* 2H). Submaximal (at 25%, 50%, and 75% maximum wattage achieved at initial CPET) heart rates were also significantly lower following HIIT (25%: −5 beat·min^−1^, *P* < 0.05; 50%: −3 beat·min^−1^, *P* = 0.05; 75%: −5 beat·min^−1^, *P* < 0.01).

### Mitochondrial oxidative phosphorylation capacity

**Figure 3:**
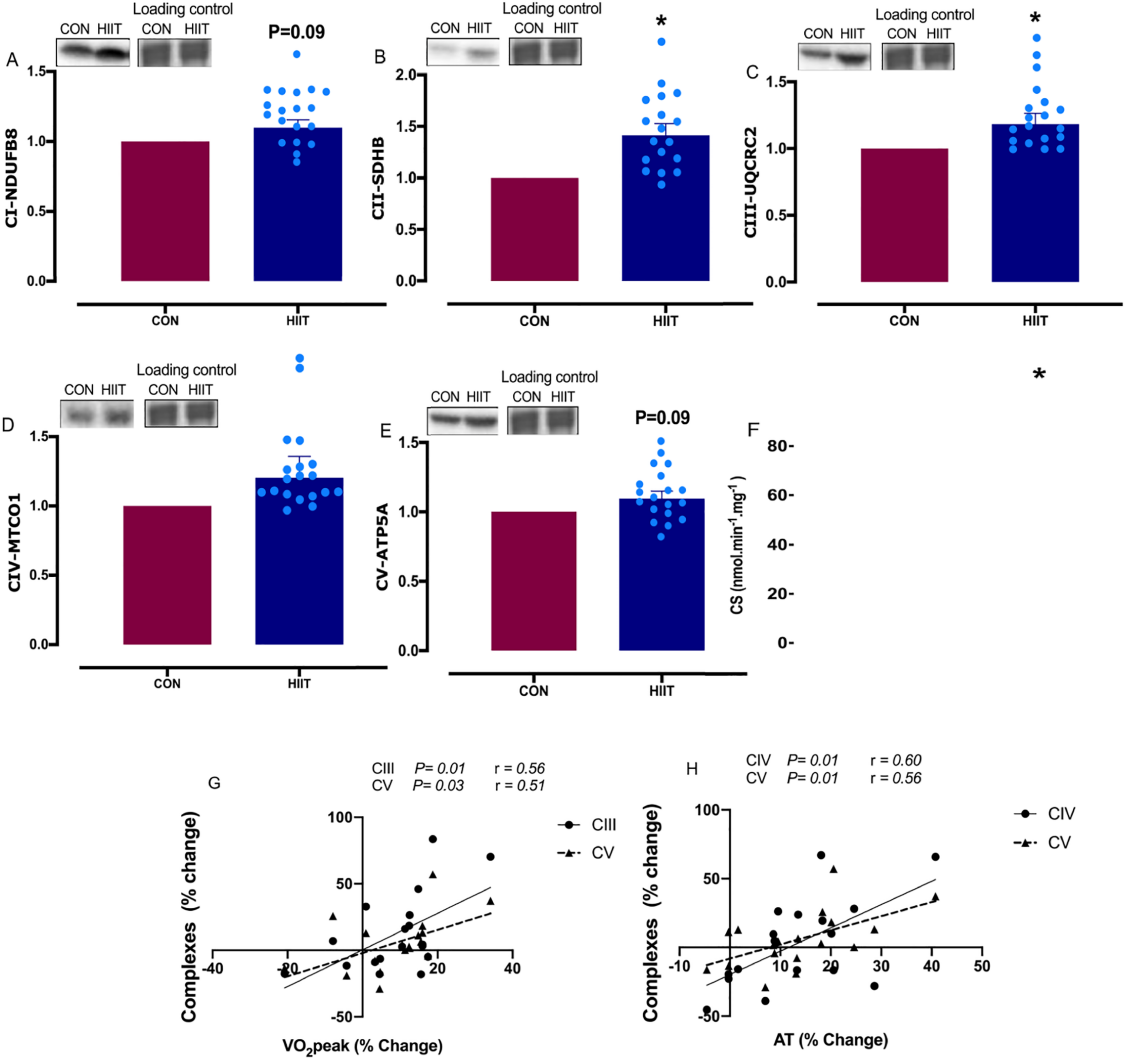
Markers of mitochondrial oxidative phosphorylation amounts after a control period (CON) or high‐intensity interval training (HIIT). *(A)* NADH dehydrogenase (ubiquinone) 1β subcomplex 8 (NDUFB8; C‐I); *(B)* succinate dehydrogenase subunit B, SDHB (*C‐II*); *(C)* ubiquinol‐cytochrome *c* reductase core protein 2 (UQCRC2; C‐III); *(D)* mitochondrial cytochrome *c* oxidase subunit 1 (MTCO1; C‐IV); *(E)* ATP synthase subunit 5α (ATP5A; C‐V); *(F)* citrate synthase (CS) activity; *(G)* and *(H)* correlation between change in mitochondrial complexes and cardiorespiratory fitness measures changes. Values are means ± SEM. Analysis via paired Student's *t* tests and Pearson's correlation coefficient. *P < 0.05. vs. CON. Data are accompanied by representative blots and a typical Coomassie stain.

The HIIT enhanced protein levels of OxPhos complex (C)II (1.4 ± 0.1‐fold change, *P* = 0.002) and CIII (1.2 ± 0.08‐fold change, *P* = 0.03), with a numerical increase in CI (1.1 ± 0.05‐fold change, *P* = 0.09) and CV (1.1 ± 0.05‐fold change, *P* = 0.09) (*Figure* 3A–3E). HIIT also augmented mitochondrial density (estimated by changes in CS activity: 52.4 ± 4 to 67.9 ± 5.1 nmol·min^−1^·mg^−1^, *P* = 0.005; *Figure* 3F), with a significant correlation between increases in CIII (*r* = 0.56, *P* = 0.01) and CV (*r* = 0.51, *P* = 0.03; *Figure* 3G) and changes in VO_2_ peak and also between increases in CIV (*r* = 0.60, *P* = 0.01) and CV (*r* = 0.56, *P* = 0.01; *Figure* 3H) and changes in AT.

### Body composition and muscle architecture

**Figure 4:**
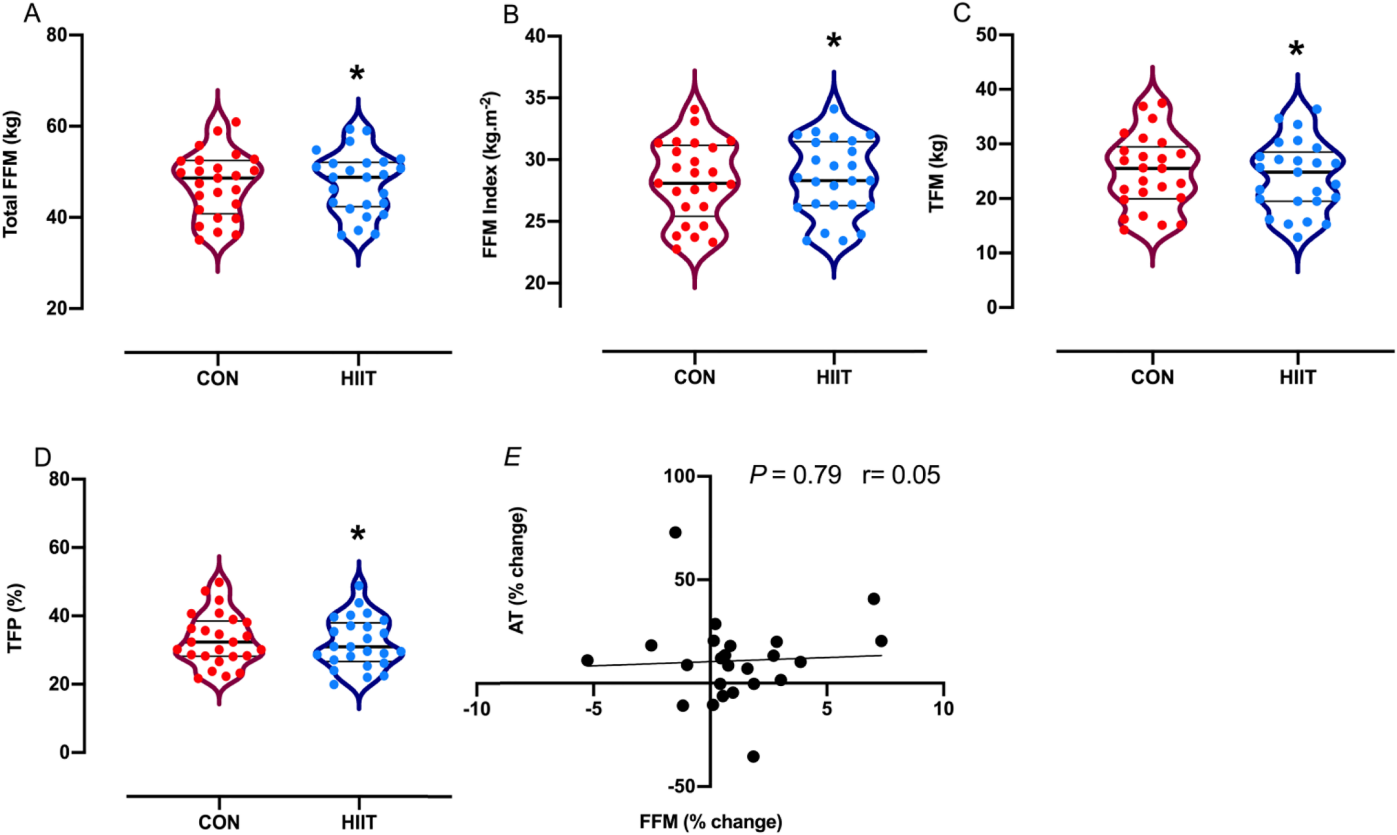
*(A–D)* Violin plots represent a full distribution of raw data (dots), median (central line), and interquartile range (lower and upper lines) of body composition after a control period (CON) and after high‐intensity interval training (HIIT). *(E)* Correlation between fat‐free mass (FFM) and anaerobic threshold (AT) changes with HIIT. Abbreviations: TFM, total fat mass; TFP, total fat percentage. Analysis via paired Student's *t* tests. * *P* < 0.05 vs. CON.

Significant increases in total FFM (47.2 ± 1.4 to 47.6 ± 1.3 kg, *P* = 0.04; *Figure* 4A), FFM index (28.2 ± 0.6 to 28.5 ± 0.6 kg·m^−1^, *P* = 0.03; *Figure* 4B) and reductions in TFM (24.8 ± 1.3 to 24 ± 1.2 kg, *P* = 0.0002; *Figure* 4C) and TFP (33.1 ± 1.5 to 32.1 ± 1.4%, *P* = 0.0008; *Figure* 4D) were observed after HIIT, with no correlation between changes in AT and changes in FFM (*r* = 0.05, *P* = 0.79, *Figure* 4E). Measures of local skeletal muscle remodelling (MT, FL, and PA) did not change during either period (i.e. CON or HIIT) (*Table* 2).

**Table 2:** Muscle architecture measures

Parameter	Assessment		*P* value	Assessment	*P* value
	Baseline	CON		HIIT	
Muscle thickness (cm)	1.9 ± 0.1	1.9 ± 0.1	0.92	2 ± 0.1	0.25
Fascicle Length (cm)	7.2 ± 0.2	7.1 ± 0.2	0.75	7.3 ± 0.2	0.34
Pennation angle (°)	13.4 ± 0.4	13.8 ± 0.5	0.92	14.2 ± 0.4	0.46

Abbreviations: CON, after a 4 week no‐intervention control period; HIIT, after a 4 week high‐intensity interval training period.

Values are mean ± SEM.

### Muscle protein synthesis

**Figure 5:**
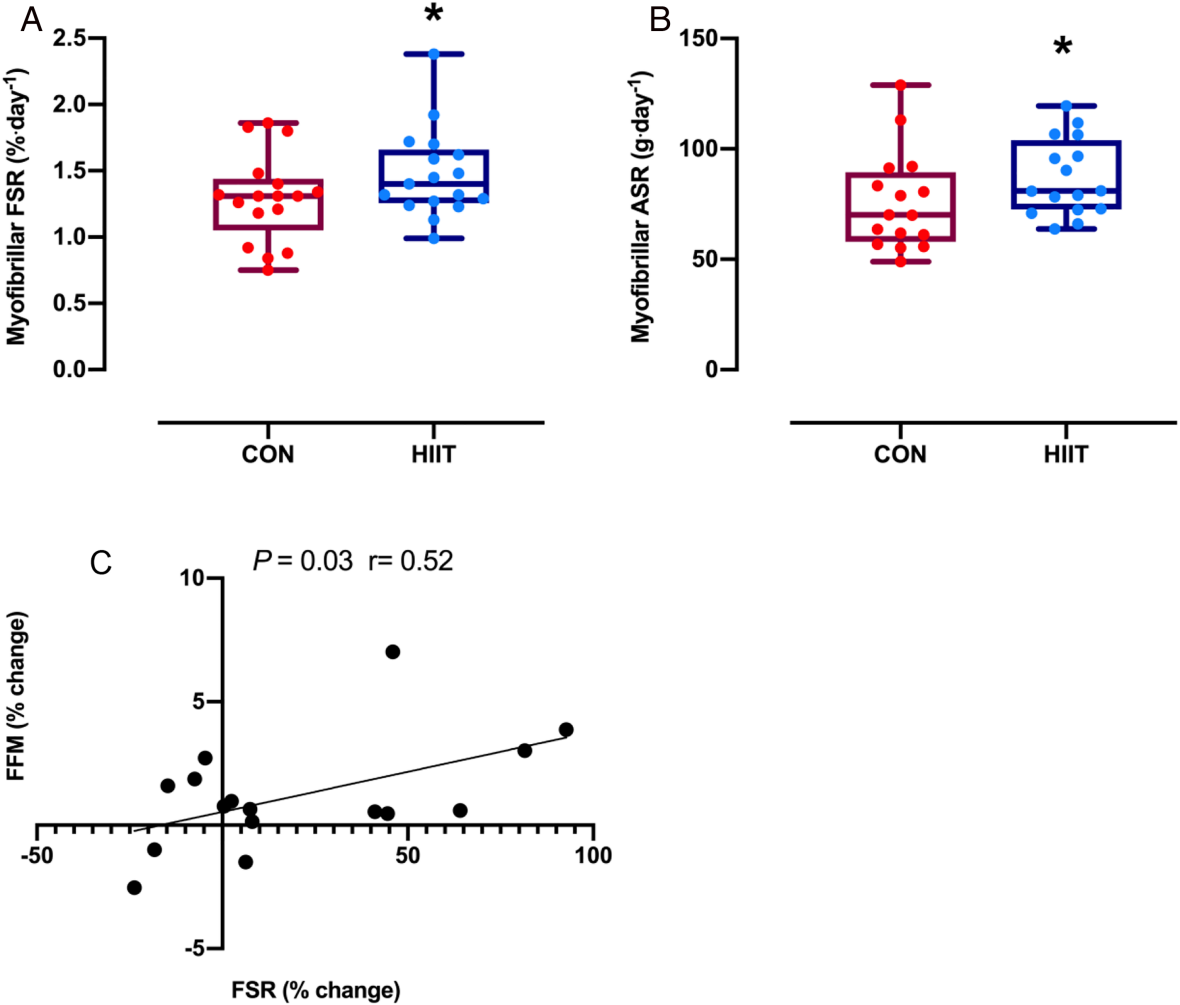
Box plot of *(A)* fractional synthesis rate (FSR) and *(B)* absolute synthetic rate (ASR) during a control period (CON) and high‐intensity interval training (HIIT). *(C)* Correlation between fat‐free mass (FFM) and FSR changes with HIIT. Distribution of raw data (dots) are represented as median (central line), interquartile range (box margins), and adjacent values (whiskers). Analysis via paired Student's *t* tests and Pearson's correlation coefficient. *P < 0.05 vs. CON.

The MPS was significantly higher during HIIT than CON (1.3 ± 0.1 to 1.5 ± 0.1%·day^−1^, *P* = 0.03; *Figure* 5A). Furthermore, absolute synthetic rate was significantly increased after HIIT (75.7 ± 5.5 to 87.1 ± 4.3 g·day^−1^, *P* = 0.03; *Figure* 5B). There was a significant correlation between changes in MPS and FFM gains (*r* = 0.52, *P* = 0.03; *Figure* 5D).

### Mechano‐signals and anabolic/catabolic signalling

**Figure 6:**
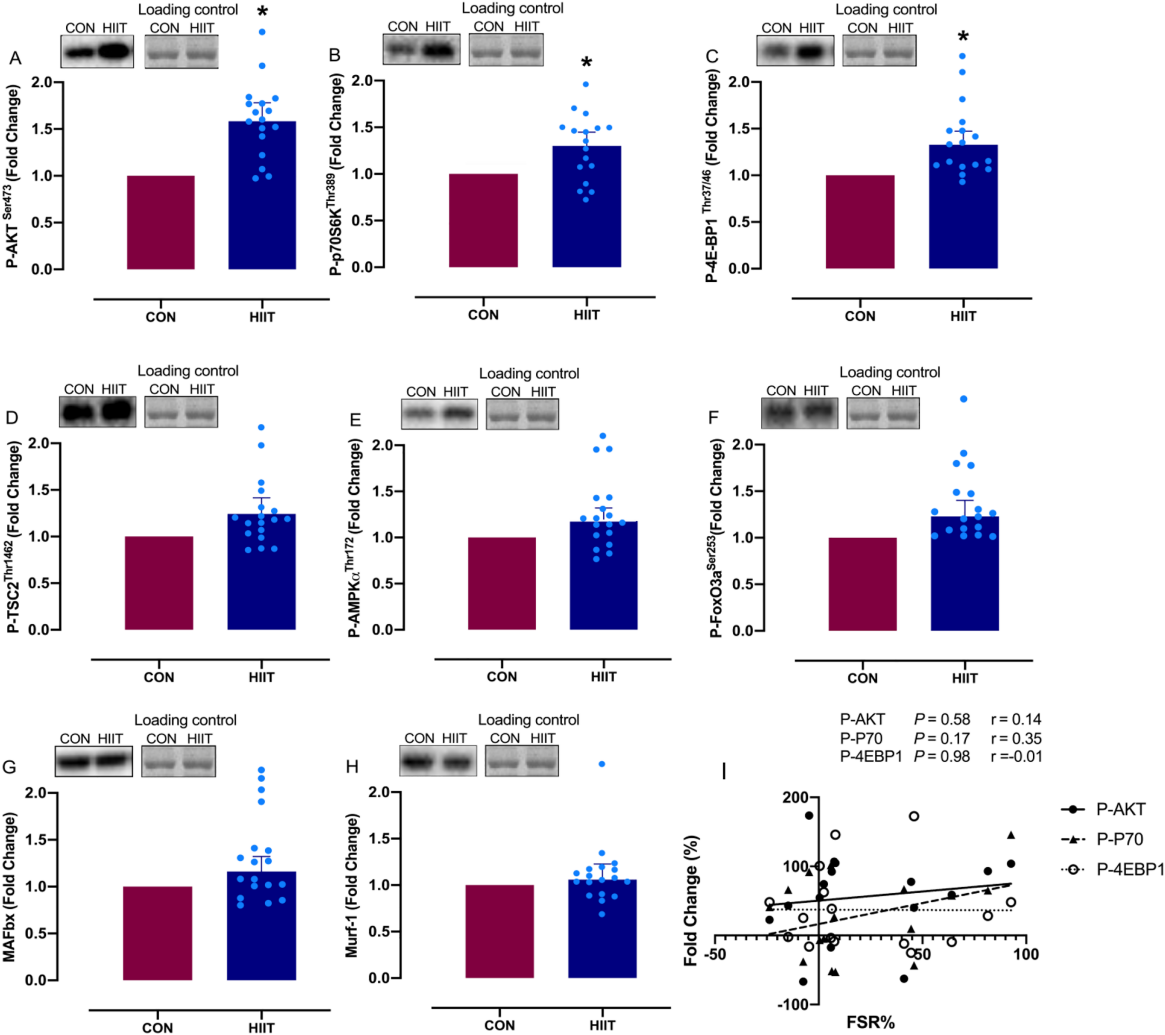
*(A–H)* Changes in intramuscular signalling pathways before and after 4 weeks high‐intensity interval training (HIIT). *(I)* Correlation between change in phosphorylation and fractional synthesis rate (FSR) changes with HIIT. Values are means ± SEM. Analysis via paired Student's *t* tests and Pearson's correlation coefficient. * *P* < 0.05 vs. CON. Data are accompanied by representative blots and a typical Coomassie stain.

Phosphorylation of AKT^Ser473^ (1.6 ± 0.1‐fold change, *P* = 0.004; *Figure* 6A), p70S6K^Thr389^ (1.3 ± 0.1‐fold change, *P* = 0.02; *Figure* 6B) and 4EBP1^Thr37/46^ (1.3 ± 0.1‐fold change, *P* = 0.01; *Figure* 6C) was increased after HIIT. Conversely, there were no significant changes in phosphorylation of tuberin/TSC2^Thr1462^, forkhead box O3 (FoxO3a)^Ser253^, AMPKα^Thr172^, MAFbx, or Murf‐1 after HIIT (*Figure* 6D–6H).

### Habitual activity levels

Based on our acceptance criteria of >80% of minute‐by‐minute physical activity data needing to be available for a 24 h recording to be accepted, 12 subjects were included in this analysis.^28^ Out with the HIIT sessions, there was no significant difference in the number of activity counts per day during CON vs. HIIT (19 367 ± 7824 vs. 17 592 ± 8677, *P* = 0.23), nor in the amount of time spent at various metabolic equivalent (MET) levels (min/day)^34^ (all CON vs. HIIT: <1.5 METs: 1194 ± 131 vs. 1201 ± 163; 1.5 to 3 METs: 128 ± 66 vs. 121 ± 88; 3 to 6 METs: 29 ± 36 vs. 46 ± 66; 6 to 10.2 METs: 0.26 ± 0.62 vs. 0.21 ± 0.46; >10.2 METs: 0.31 ± 0.98 vs. 0.02 ± 0.04, all *P* > 0.05) suggesting that observed changes over the HIIT period were due HIIT per se and not changes in habitual physical activity.

### Quality of life

The HIIT increased self‐rated health status using the 5EQ‐5DL‐5L questionnaire (85 [80–90] to 90 [81.25–90], *P* < 0.05) with no significant change across CON. There was no change in weighted health state index during CON or HIIT.

## Discussion

No study had previously examined the effect of HIIT in relation to both FFM and CRF and associated mechanisms in octogenarians with comorbidities in both sexes. Herein, we report that just 4‐weeks of HIIT positively modulated CRF and FFM, ostensibly via enhancing protein accretion and mitochondrial bioenergetics; thereby offering a potential strategy to offset two well‐established age‐related physiological deteriorations in octogenarians with diseases.

Cardiorespiratory fitness is the culmination of VO_2_ peak, AT, and exercise capacity and is an attribute known to decrease the risk of morbidity and mortality^4^; as such, we determined the role of HIIT in relation to aspects of CRF and established that HIIT augmented AT, VO_2_ peak, and exercise capacity. In previous work, Sloth *et al*.,^35^ reported that short‐term, low‐volume HIIT ranging from 2 to 8 weeks in duration was effective for improving VO_2_ peak (∼4–13%) in healthy sedentary and recreationally active adults, while HIIT for 4 weeks augmented VO_2_ peak (9.6%) and AT (13%) in healthy older adults.^24^ Similarly, in our study, AT was augmented (10%) over 4 weeks HIIT; which has been shown to be a good predictor of survival in patients with comorbidities, with a value of <11 mL·kg^−1^·min^−1^ considered the threshold for classifying surgical patients as high risk of mortality.^5^ These results are potentially indicative of wider implications for long‐term healthy ageing as improving CRF is associated with a lower risk of all‐cause and CVD mortality.^36^ In addition to improving CRF components, our HIIT protocol also reduced blood pressure. Prior to this study, the effect of short‐term HIIT on blood pressure was inconclusive^24^; nevertheless, we have now provided supporting evidence that in older individuals with disease short‐term HIIT reduces systolic blood pressure, which is associated with lower long‐term life‐threatening risk, that is, from total mortality and stroke.^37^

In assessing muscle‐level mechanisms underlying augmentation in CRF, we found that HIIT increased mitochondrial content and aspects of proteostasis even in our ‘very’ old comorbid adults. Muscle mitochondria are subject to age‐related remodelling; for instance, ageing is associated with decreases in mitochondrial biogenesis and oxidative capacity^38^ and mitochondrial enzyme activity (e.g. CS), resulting in impaired mitochondrial function^39^ (especially in individuals older than 80 years suffering from severe muscle loss, chronic diseases, or orthopaedic issues^40^). Exercise, including HIIT, is well known to induce mitochondrial adaptations. For instance, Broskey et al., showed that trained older individuals had significantly greater mitochondrial content and function, which correlated with exercise efficiency.^41^ Further, HIIT for 2 weeks increased CS activity and mitochondrial protein C‐IV abundance,^42^ while longer term (6 weeks) HIIT increased VO_2_ peak, CS activity, and C‐IV abundance in younger (<65 years) adults.^15^ In older adults, 12 weeks HIIT increased the abundance of mitochondrial C‐I, II, IV, and V and CS in older adults 65–76 years^43^ and those >73 years^44^; however, these individuals were free from disease. Given these and other studies (e.g. 16 weeks traditional endurance training increased VO_2_ peak and CS activity^45^), there is clearly a robust relationship between CRF and muscle mitochondrial density.^46^ In our study, HIIT augmented CS activity and mitochondrial protein C‐II and C‐III, with a trend towards an increase in C‐I (important component of longevity and health^47^) and C‐V. C‐I improvements alone account for approximately half of all augmentations in OxPhos,^47^ with OxPhos capacity important in preventing the accumulation of free radicals and damaged organelles that could negatively impact muscle function, particularly in ageing.^16^ Therefore, in line with other studies,^48^ we have shown that HIIT is a potent intervention for the up‐regulation of cellular oxidative capacities which led to an increase in CRF^49^ in octogenarians with disease.

Given established links between ageing and anabolic resistance (to both nutrition and exercise)^50^ and subsequent muscle loss known to lead to long‐term health challenges,^51^ we postulated that short‐term HIIT would lead to increases in whole‐body FFM via altered muscle protein metabolism and decreases in TFM in comorbid octogenarians. Prior work demonstrated the ability of 3 months HIIT to impact multiple body composition components (e.g. 1% increase in FFM and 5% decrease in TFM) in both pre‐menopausal and post‐menopausal women.^52^ Consistent with this thesis, HIIT has been shown to increase FFM (1–3%) in healthy young and older adults (20–75 years),^9^, ^10^, ^11^ with the highest increases in younger cohorts; indicating diminishing adaptation with ageing.^50^ Nonetheless, the efficacy of this training mode in those with comorbidities had not previously been investigated. The physiological impact of comorbidities (e.g. reduced anabolic responses, increased net muscle protein breakdown, and associated FFM losses^1^, ^53^), and that in older adults this insult is adjuvant to the detrimental processes of advanced chronological ageing blunting the efficacy of exercise training^54^ (including HIIT^1^), may explain the attenuated (albeit notable and significant) increases in FFM (∼0.8%), in our study.

In assessing the mechanisms underlying the anabolic effects of HIIT in octogenarians with disease, we demonstrated that HIIT augments MPS.^1^ The logical extrapolation of a sustained increase in net muscle protein deposition after HIIT is a modest increase in FFM and also VO_2_ peak,^14^ entirely in line with our body composition and CRF data. In support of these findings, although based on acute (up to 24 h) and not chronic measures of MPS, a single HIIT session has previously been shown to increase MPS in healthy ∼65 years men,^14^ with further supportive work in pre‐clinical animal models, for example, 8 weeks HIIT upregulated MPS and related signalling pathways in middle‐aged rats.^13^ As such, there was already some evidence of physiological adaptations to HIIT falling somewhere between resistance and aerobic training on the ‘exercise spectrum’, that is, HIIT stimulates adaptations traditionally associated with both aerobic and resistance exercise training.^14^ Hereby, our results demonstrate the efficacy of this training mode to elicit net protein accretion, even in comorbid octogenarians with disease, via elevated chronic MPS. In order to explore the mechanisms underpinning increases in MPS, we investigated aspects of translational ‘efficiency’.^55^ It is noteworthy from past work that activation of these molecular transducers is blunted in older age^28^, ^50^ and that in the present study, HIIT was able to repeal these impairments. In support of this, HIIT augmented Akt–mTOR signalling‐activity (which regulates MPS^1^),^13^ presumably explaining enhanced FFM gains and suggesting that HIIT is a potent stimulus for inducing anabolic responses in skeletal muscle via increasing the efficiency of signal transduction.^56^ Supporting this notion, anabolic signalling proteins^57^ after 4 weeks HIIT remained elevated ∼72 h after the final HIIT session when compared with before training, as has been reported previously.^58^ This prolonged pro‐anabolic signalling profile may explain the increases in FFM observed in this study.

We do acknowledge some limitations to our study. First, our DXA‐derived data showing small changes in FFM must be interpreted with caution given the imprecision of DXA as a tool to measure muscle mass based on the DXA assumption that all non‐fat and non‐bone FFM is skeletal muscle mass.^59^, ^60^ Further, as protein intake > the recommended daily allowance increases muscle strength and mass in older individuals,^61^, ^62^ and that amino acid sufficiency is required for optimal hypertrophic responses to exercise training,^63^ the lack of dietary intake data is a further limitation. There is potential that adjuvant protein nutrition, in those with low intake, may potentiate favourable adaptations to HIIT, and this should be explored in future studies.

## Conclusions

Short‐term/low‐volume HIIT is an effective intervention in very old individuals with comorbidities. Our short‐term HIIT regime was well‐tolerated and could be useful in peri‐operative and pre‐/rehabilitation settings in octogenarians with comorbidities, with no adverse safety events occurring in this study.^64^ This adds to the evidence that HIIT is safely deliverable to people with disease^2^, ^6^ and in older age^43^ and is in keeping with data from large‐scale studies using HIIT in cardiac rehabilitation.^2^ Finally, we revealed the likely mechanisms underlying the effect of HIIT in relation to ‘overcoming’ aspects of age‐related anabolic resistance which result in an improvement in CRF and body composition, vis‐à‐vis, elevating MPS, enhancing translational efficiency, and improving cellular oxidative capacities. We propose our HIIT protocol should now be subject to larger trials with direct clinical outcome endpoints.

## Funding

This research was supported by the MRC Versus Arthritis Centre for Musculoskeletal Ageing Research (grant numbers MR/P021220/1 and MR/R502364/1), the Dunhill Medical Trust (R468/0216) and the National Institute for Health Research Nottingham Biomedical Research Centre.

## Conflict of interest

The authors have declared that no conflict of interest exists.

## Supporting information


**Table S1:** HIIT Acceptability Questionnaire
