# Supplementary material for: The physiological impact of high‐intensity interval training in octogenarians with comorbidities
Source: J Cachexia Sarcopenia Muscle. 2021 May 31;12(4):866–79. doi: 10.1002/jcsm.12724 (PMC8350218; doi:10.1002/jcsm.12724)
Supplement: Supplementary file 1 — Table S1: HIIT Acceptability Questionnaire [file JCSM-12-866-s001.docx]

**Supplementary Information**

***HIIT acceptability***

Our HIIT protocol was deemed highly acceptable by this cohort (Table S1) with 89% of individuals ‘strongly agreeing’ that HIIT was enjoyable. A similar number of participants reported that they would ‘do HIIT again’ (86% ‘strongly agree’) and 79% would ‘recommend HIIT to others’. When asked: ‘What was your favourite part of HIIT?’ 45% of participants specifically stated ‘social interaction with the staff’.

# **Table S1: HIIT Acceptability Questionnaire**

| Comment (n=28) | Median score (range)  5 – Strongly agree  1 – Strongly disagree |
| --- | --- |
| HIIT was well explained | 5 (5) |
| I enjoyed HIIT | 5 (5) |
| HIIT was a time burden | 1 (1-4) |
| I would recommend HIIT to others | 5 (5) |
| HIIT was more demanding than expected | 3 (2-5) |
| I would do HIIT again | 5 (5) |
| The travelling involved with HIIT interfered with my life | 1 (1-3.5) |
| The physical strain of HIIT interfered with my life | 1 (1-2.5) |
| I believe my fitness has improved | 5 (4-5) |
| I would like to have exercised in a group | 1 (1-3) |
| I would like to have exercised at home | 1 (1) |

Data from HIIT acceptability questionnaire completed by participants following HIIT protocol completion. 5-point Likert scale data represented as Median (range).

***Participant engagement***

Of 32 participants recruited to this study, 28 completed all assessment sessions and HIIT. One participant voluntarily withdrew after the death of their spouse. One participant did not like the feeling in his legs induced by HIIT and voluntarily withdrew after one session. One volunteer was unable to cycle on the static ergometer due to knee pain (known osteoarthritis) and therefore did not undergo the initial CPET to begin the study. The last participant to not complete was withdrawn from the study as he was started on a beta-blocking agent following advice from a cardiologist. This patient had well-documented cardiovascular disease (coronary artery bypass graft, abdominal aorta repair and hypertension) and breached our safety criteria of a >2mm ST depression in V5-6 during the second half of the HIIT programme (session 7) following an increase in the intensity. He was asymptomatic throughout and tolerated HIIT well despite this finding.
